# Genome-Wide Identification of the *LEA* Gene Family in *Myricaria laxiflora* and Its Responses to Abiotic Stress

**DOI:** 10.3390/genes16070763

**Published:** 2025-06-29

**Authors:** Di Wu, Tonghua Zhang, Linbao Li, Haibo Zhang, Yang Su, Jinhua Wu, Junchen Wang, Chunlong Li, Guiyun Huang

**Affiliations:** 1 Yangtze River Biodiversity Research Centre, China Three Gorges Corporation, Wuhan 443133, China; wu_di3@ctg.com.cn (D.W.); li_linbao@ctg.com.cn (L.L.); zhang_haibo@ctg.com.cn (H.Z.); su_yang@ctg.com.cn (Y.S.); wu_jinhua@ctg.com.cn (J.W.); wang_junchen@ctg.com.cn (J.W.); 2 Hubei Key Laboratory of Rare Resource Plants in Three Gorges Reservoir Area, Yichang 443100, China; 3 National Engineering Research Center of Eco-Environment Protection for Yangtze River Economic Belt, China Three Gorges Corporation, Wuhan 100083, China; 4 National Key Laboratory for Germplasm Innovation & Utilization of Horticultural Crops, College of Horticulture and Forestry Sciences, Huazhong Agricultural University, Wuhan 430070, China; zhangtonghua@mail.hzau.edu.cn (T.Z.); cl2444@mail.hzau.edu.cn (C.L.)

**Keywords:** *Myricaria laxiflora*, *LEA* gene family, abiotic stress, *cis*-regulatory elements, flooding adaptation

## Abstract

Background: The *late embryogenesis abundant* (*LEA*) gene family plays a critical role in abiotic stress tolerance during plant growth and development. Myricaria laxiflora, as a key pioneer species in the extreme hydrological fluctuation zone of the Yangtze River, has evolved unique adaptation mechanisms potentially linked to gene family evolution. However, the molecular mechanisms underlying how the *LEA* gene family responds to alternating flooding–drought cycles remain unclear. Methods and Results: In this study, we identified 31 *LEA* genes through whole-genome and transcriptome analyses using bioinformatics approaches, and classified them into nine subfamilies based on protein sequence similarity. These genes were distributed across 12 chromosomes. Our analysis revealed that *LEA* promoters contain cis-acting elements associated with anaerobic induction, abscisic acid (ABA) response, and combined low-temperature/light stress, suggesting their role in a multi-tiered environmental signal integration network. Spatio-temporal expression profiling further indicated that root-specific *LEA* genes maintain cellular integrity via membrane lipid binding, while leaf-predominant members cooperate with the antioxidant system to mitigate photoinhibition damage. Conclusions: This study elucidates the dynamic regulatory mechanisms of the *LEA* gene family during flooding-drought adaptation in *M. laxiflora*, providing molecular targets for ecological restoration in the Yangtze River Basin.

## 1. Introduction

As an endemic species in the Yangtze River Basin, *Myricaria laxiflora* (Tamaricaceae) is the flagship species of fragile habitats in this area. Its natural population is concentrated in the water-level-fluctuating zone of the main stream and tributaries of the Yangtze River (altitude of 145–175 m). It will suffer from 8 months of submergence and serious sediment erosion and deposition in a year. By relying on floods to achieve seed dispersal and germination, *M. laxiflora* can rapidly colonize the new tidal flat after the seasonal floods subside and become a constructive species of pioneer plant communities in the hydro-fluctuation belt [1,2].

*M. laxiflora* shows unique morphological and physiological adaptation characteristics: scaly leaves can reduce transpiration, and well-developed aerenchyma roots support oxygen diffusion during submergence. These characteristics make *M. laxiflora* play a key role in retaining sediments, enriching heavy metals, and providing microhabitats for endemic animals (such as the Chinese merganser). The ecological monitoring after the completion of the Three Gorges Dam shows that *M. laxiflora* has irreplaceable ecological functions in maintaining biodiversity and sediment retention in the reservoir area [3,4,5]. The Three Gorges Project (impoundment in 2003) caused about 70% of the native habitats of *M. laxiflora* to be permanently submerged, and the remaining populations were fragmented and distributed in the tributary hydro-fluctuation belt, facing the loss of genetic diversity and renewal obstacles due to the change in flood rhythm. Currently, *M. laxiflora* has been listed as an endangered (EN) species by the International Union for Conservation of Nature (IUCN) and included in China’s List of National Key Protected Wild Plants (Class II) [6].

Since 2010, China has launched ex situ conservation initiatives (e.g., the Wuhan Botanical Garden Germplasm Repository) and in situ restoration trials. Genomic research has identified candidate genes associated with flooding stress response in *M. laxiflora* [7,8], yet practical bottlenecks remain, including the low survival rate of transplanted seedlings in hydro-fluctuation zones and the unclear coupling mechanism between reservoir operations and population dynamics.

Late embryogenesis abundant (LEA) proteins constitute a class of small hydrophilic proteins ubiquitously present in plants. Their nomenclature derives from high abundance during late seed development and enrichment under dehydration conditions [9]. Based on conserved domain features, the LEA protein family is classified into seven subfamilies (LEA_2, LEA_3, and LEA_5). Notably, LEA_2 (dehydrins) and LEA_3 (seed maturation proteins) are extensively studied due to their broad involvement in stress responses [10]. Since initial isolation from cotton embryos in the 1980s, this protein family has been demonstrated to play critical roles in plant tolerance to abiotic stresses, including drought, high salinity, low temperature, and oxidative stress [11]. Advances in genomics and molecular biology have propelled research on LEA proteins’ functional mechanisms, evolutionary traits, and potential applications in enhancing crop stress resistance. For instance, *SlLEA6* knockout in tomato compromised antioxidant defenses and reactive oxygen species (ROS)-scavenging systems, thereby reducing cellular damage resistance and drought tolerance [12]. Heterologous expression of the ginseng *PgLEA2-50* gene in tobacco enhanced osmotic regulation capacity and antioxidant activity in transgenic lines [13].

However, current research still faces challenges; the functional conservation and differentiation mechanisms of LEA proteins in *M. laxiflora*’s response to flooding stress remain unclear. To address this knowledge gap, this study employs bioinformatics approaches to identify the *LEA* gene family through a genome-wide analysis of *M. laxiflora*. We further characterize the sequence features and potential biological functions of these genes, thereby establishing a theoretical foundation for investigating the *LEA* gene family’s role in abiotic stress responses.

## 2. Materials and Methods

### 2.1. Plant Material and Experiment Design

The experimental materials comprised uniformly grown 1-year-old seedlings of identical genetic background, cultivated at the experimental farm of the Yichang Yangtze River Rare Plant Research Institute, China (March 2020). The seedlings were subjected to flooding in test pools under ambient temperatures averaging 19–27 °C daily. Root samples were collected at 0, 6, 12, 18, 24, 30, 36, and 48 h post-flooding initiation. Following treatment, the seedlings were transferred to control conditions for 12 h recovery (RR12) with subsequent root sampling. All samples were flash-frozen in liquid nitrogen and stored at −80 °C pending analysis [7].

### 2.2. Search and Identification of LEA Gene Sequence in M. laxiflora

To identify *MlLEA* genes, whole-genome sequencing data on *M. laxiflora* were obtained from Figshare (Dataset ID: 25375366). Candidate sequences were screened using the Pfam database to detect LEA-conserved domains, followed by a removal of sequences lacking these domains. The resulting *MlLEA* genes were submitted to NCBI. *AtLEA* reference sequences were retrieved from The Arabidopsis Information Resource (TAIR) and NCBI. Finally, protein physicochemical properties were analyzed via ExPASy ProtParam, while subcellular localization predictions were performed using Cell-PLoc 2.0.

### 2.3. Nomenclature and Phylogenetic Tree Analysis of LEA Family Genes in M. laxiflora

Based on the conserved domain architecture, the LEA protein family was classified into subfamilies with a systematic nomenclature reflecting the subfamily characteristics. Reference protein sequences of *Arabidopsis thaliana LEA* family members were retrieved from TAIR. Phylogenetic analysis was then conducted using MEGA7.0 for tree construction and the iTOL platform for visualization, comparing the LEA proteins from *A. thaliana* and *M. laxiflora*.

### 2.4. Conserved Domain and Gene Structure Analysis and Cis-Element Analysis of LEA Gene in M. laxiflora

Conserved motifs in LEA proteins were predicted using the MEME suite with the motif count set to 10 and default parameters. The motif distributions were integrated with the *M. laxiflora LEA* gene phylogeny and visualized via TBtools (v2.030). Chromosomal locations were mapped using the *M. laxiflora* genome annotation files. Promoter regions (defined as 2 kb upstream of the initiation codon ATG) were extracted from the genomic data using TBtools (v2.030) and analyzed in PlantCARE for *cis*-acting element identification. Elements were filtered to retain abiotic stress-responsive *cis*-regulatory elements, excluding unannotated/blank entries.

### 2.5. Chromosomal Mapping, Gene Duplication, and Collinearity Analysis

Chromosomal locations of *M. laxiflora LEA* genes, including chromosome numbers, gene start/stop positions, and chromosome lengths, were extracted from the genome annotation files. Using TBtools (v2.030), the intragenomic collinearity within *M. laxiflora* and the interspecific collinearity between *M. laxiflora* and *A. thaliana* genomes were analyzed.

### 2.6. Differential Expression Analysis of LEA Gene Family in Myricaria laxiflora

Total RNA extraction, library construction, and RNA-seq were conducted by Shanghai OE Biotech Co., Ltd. (Shanghai, China). RNA integrity was assessed with an Agilent 2100 Bioanalyzer (Santa Clara, CA, USA). Libraries were prepared with the TruSeq Stranded mRNA LT Kit (Illumina, CA, USA) and sequenced on an Illumina HiSeq 2500 platform in 150 bp paired-end mode. Three biological replicates were included per sample.

Clean reads were generated by processing raw data with Trimmomatic to remove adapters and low-quality sequences. De novo transcriptome assembly was performed using Trinity with paired-end reads. The longest isoform per gene locus was designated as the unigene based on transcript length for downstream analysis. Gene expression levels were quantified with FPKM (fragments per kilobase of transcript per million mapped reads) [14,15].

### 2.7. Statistical Analysis

Differentially expressed genes (DEGs) were identified using DESeq2 with thresholds of false discovery rate (FDR) < 0.05 and |log_2_ (fold change)| > 1. Differentially expressed proteins (DEPs) were detected by Student’s *t*-test with |fold change| > 2 and *p*-value < 0.05. Three biological replicates were analyzed per sample. Protein–gene expression correlations were assessed via Pearson’s correlation coefficient.

## 3. Results

### 3.1. Identification of LEA Gene in M. laxiflora

**Figure 1:**
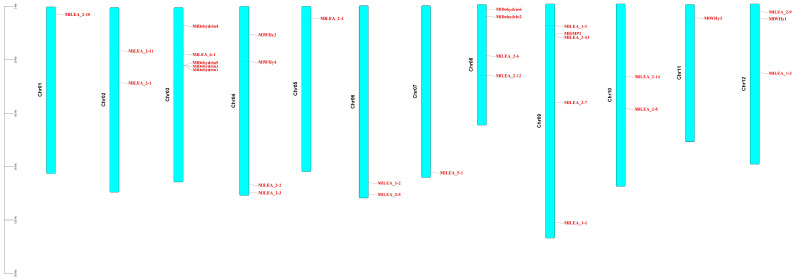
Chromosomal localization of LEA gene family in *Myricaria laxiflora*.

Chromosomal localization of 31 LEA proteins was mapped using *M. laxiflora* gene annotation files in TBtools. Analysis revealed scattered gene distribution across chromosomes (Figure 1), with 1–5 genes per chromosome. Tandem gene clusters were observed in specific subfamilies, notably dehydrins, showing localized distribution on chromosomes 3 and 8.

### 3.2. Analysis of Structural Domains and Physicochemical Properties of LEA Gene Family Members in M. laxiflora

Using Clustal W, we performed a multiple sequence alignment, which revealed conserved domains and enabled classification of the *LEA* genes into nine subfamilies: Dehydrin, LEA_1, LEA_3, LEA_5, LEA_6, WHy, SMP, and LEA_2 superfamily (Table 1, Figure S1). The physicochemical properties and subcellular localization of the 31 identified *LEA* genes were analyzed (Table 2). Predicted localizations indicated that all *LEA* family members were predicted to localize to chloroplasts. The protein characteristics showed that the amino acid length ranged from 76 aa (MlLEA_6-1, MlLEA_1-3) to 420 aa (MlLEA_2-11); the molecular weight ranged from 8.08 kDa (MlLEA_6-1) to 45.91 kDa (MlLEA_2-11); and the isoelectric point (pI) ranged from 4.68 (MlWHy1) to 10.25 (MlLEA_2-6).

### 3.3. Phylogenetic Tree Analysis of LEA Gene Family Members in M. laxiflora

**Figure 2:**
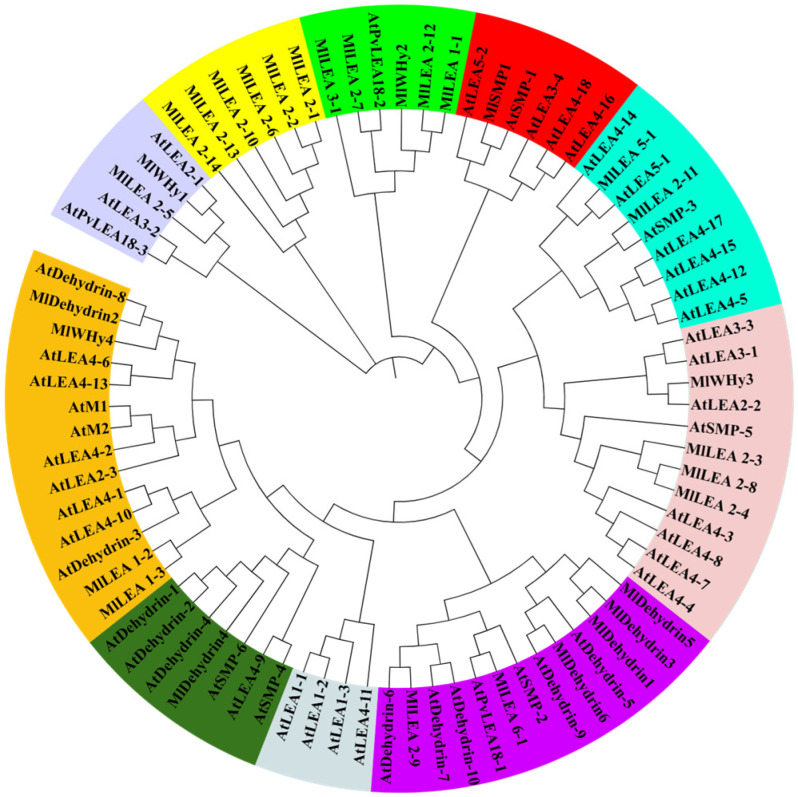
Phylogenetic tree of LEA family genes in *M. laxiflora* and *A. thaliana*. Different colors represent different groups.

A phylogenetic tree was reconstructed using MEGA7 with the 31 identified *MlLEA* genes and *AtLEA* reference sequences. Based on evolutionary relationships, the LEA family members were classified into 10 clades, each demarcated by distinct colored backgrounds (Figure 2).

### 3.4. The Domain, Motif Distribution Pattern, and Gene Structure of LEA Gene Family Members in M. laxiflora

**Figure 3:**
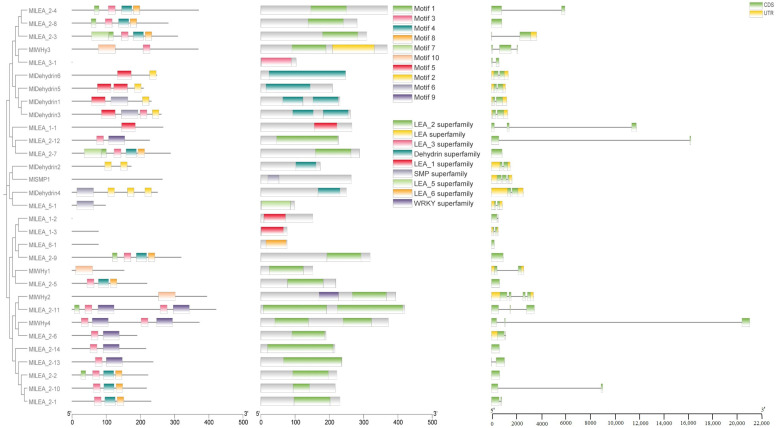
*LEA* gene evolution, conserved domain, and gene structure analysis of *M. laxiflora*.

Conserved motifs in LEA proteins typically correlate with specific functions. In this study, ten conserved motifs (motif1–motif10) were identified in *M. laxiflora* LEA proteins (Figure 3). An analysis revealed that these motifs exhibit subfamily-specific distribution patterns. Further characterization of the gene structures and domains demonstrated that the Dehydrin, LEA_1, LEA_2, LEA_3, LEA_5, LEA_6, and SMP subfamilies all contain stress-responsive domains, consistent with their established biological roles.

### 3.5. The Cis-Acting Elements of LEA Gene in M. laxiflora

**Figure 4:**
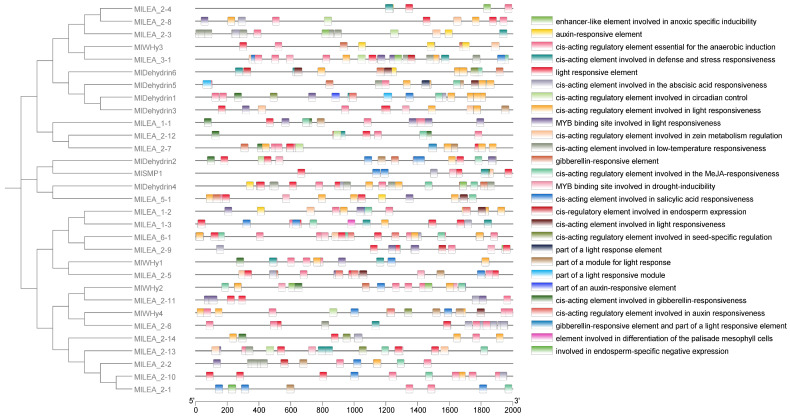
Analysis of promoter elements of LEA family genes in *Myricaria laxiflora*.

To elucidate the transcriptional regulation mechanisms of *LEA* genes, we systematically characterized *cis*-acting regulatory elements within the promoter regions of the *MlLEA* gene family in *M. laxiflora* (Figure 4) and comprehensively characterized *cis*-acting regulatory elements in the promoter regions of 31 *MlLEA* genes. A bioinformatics analysis revealed 27 functionally distinct *cis*-regulatory elements categorized into three core functional modules: (1) abiotic stress response: low-temperature responsiveness (e.g., MBS elements), anaerobic induction (e.g., ARE motifs), and drought inducibility (e.g., MYB binding sites); (2) hormone signaling: abscisic acid (ABRE), gibberellin (GARE-motif), auxin (AuxRR-core/TGA-element), jasmonic acid (CGTCA-motif) responsiveness, and salicylic acid (TCA-element) regulation; and (3) growth and development: light responsiveness (multiple G-box variants), endosperm-specific expression (GCN4_motif), palisade mesophyll differentiation *cis*-elements, and seed-specific regulation (RY-element). Notably, stress-responsive elements dominate promoter architectures, particularly in dehydrin subfamilies (e.g., MIDehydrin1-7), where >80% of genes contain ABA/drought-responsive motifs. Conversely, *MlLEA_2* and *MlWHy* members exhibit combinatorial enrichment of light-responsive and circadian control elements. These findings establish a molecular basis for *MlLEA* genes in mediating coordinated stress adaptation and developmental plasticity in *M. laxiflora*.

### 3.6. Chromosomal Distribution and Synteny Analysis of LEA Genes

**Figure 5:**
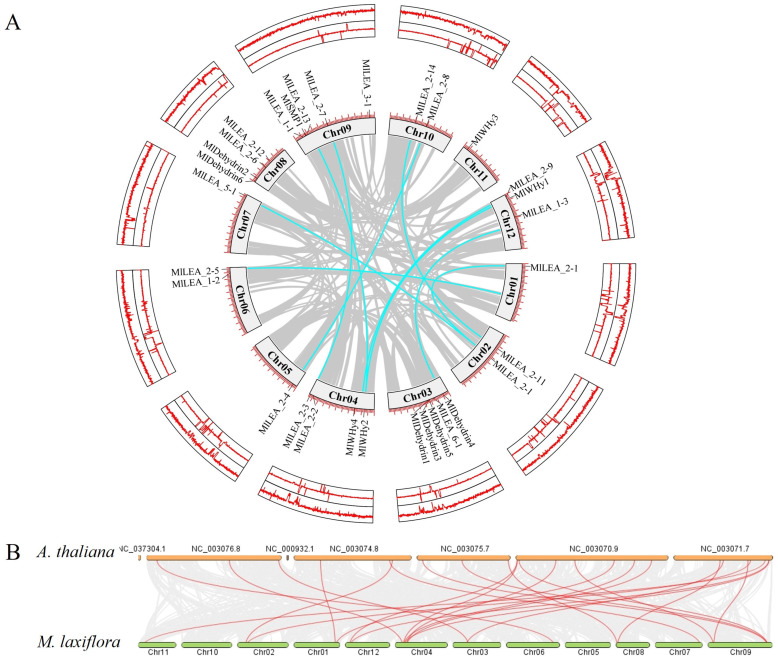
Linearization analysis of LEA family genes within and between species (*A. thaliana*) in *M. laxiflora*. (**A**) Chromosome distribution and collinearity analysis of LEA gene family in the genome of *M. laxiflora*. (**B**) Interspecific collinearity analysis of LEA gene family between *M. laxiflora* and *A. thaliana*. Note: (**B**) is the complete *Arabidopsis* genome.

*LEA* genes were unevenly distributed across 12 chromosomes, with chromosome 3 (Chr03) and chromosome 9 (Chr09) harboring the highest number (five genes each). In contrast, Chr01, Chr05, Chr07, and Chr11 contained only one gene each (Figure 5A). No significant correlation was observed between chromosome length and LEA gene density. A microsynteny analysis between *M. laxiflora* and *Arabidopsis thaliana* genomes revealed collinear blocks with a conserved gene order (Figure 5B), suggesting purifying selection on functionally critical regions. Notably, chromosomal rearrangements, including the inversion of Chr01, Chr02 and disrupted synteny in segmental regions, indicate potential evolutionary events such as chromosome fission/fusion or local inversions, providing insights into species-specific genome evolution.

### 3.7. Expression Profiling of M. laxiflora LEA Genes with RNA-seq

**Figure 6:**
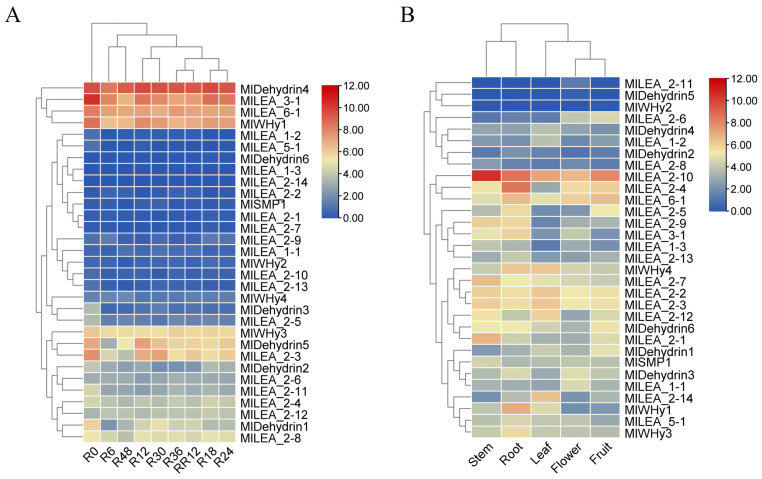
The expression pattern of LEA gene in *Myricaria laxiflora*. (**A**) The expression of LEA family genes at different times under flooding stress. (**B**) The expression pattern of LEA gene in different tissues. The data were converted to log2 (FPKM+1), and the responsiveness of the LEA gene was represented by a heat map. Blue and red represented down-regulated and up-regulated genes under different tissues and treatments, respectively. Each point represents the average of three independent biological replicates.

Transcriptomic analysis of published data revealed a high sensitivity of LEA genes in *M. laxiflora* to flooding stress. Significant differential expression of multiple *LEA* genes (e.g., *MIDehydrin4*, MILEA_3-1, MILEA_6-1, and *MWHy1y1*) was detected as early as 6 h (R6) post-flooding initiation compared to untreated controls (R0) (Figure 6A). The magnitude and pattern of this differential expression continued to evolve throughout the flooding period (R6 to R48), suggesting potential stage-specific roles for *LEA* genes in distinct phases of the stress response, including initial perception, adaptation, and maintenance. A key finding was the high reversibility of *LEA* gene expression. Following stress removal and entry into the recovery phase, the expression levels of nearly all *LEA* genes altered during flooding rapidly returned to baseline levels that were comparable to or equivalent with the R0 controls within just 12 h of recovery (RR12). This indicates that the observed expression changes are stress-specific and that the plant possesses a robust capacity for rapid transcriptional reprogramming to resume normal growth upon the cessation of the stress.

Spatiotemporal expression profiling of all 31 identified *LEA* genes (*MILEA*) in *M. laxiflora* revealed their presence across various plant organs. Transcripts for the vast majority of genes, including but not limited to *MILEA_1-1*, *MILEA_1-2*, *MILEA_1-3*, most *MILEA_2* subgroup members (*MILEA_2-1* to *MILEA_2-14*), *MILEA_3-1*, *MILEA_5-1*, *MlLEA6_1*, *MlSMP1*, *MlWHy1*, *MlWHy3*, *MlWHy4*, *MIDehydrin1-4*, and *MIDehydrin6*, were detectable at certain levels in all examined tissues (Figure 6B). This broad, non-organ-restricted expression pattern strongly suggests that *LEA* genes perform universal functions throughout the *M. laxiflora* lifecycle and in diverse fundamental physiological processes, extending beyond specific stress responses. They likely play fundamental roles in maintaining cellular homeostasis, protecting macromolecular structures, and participating in developmental regulation. In contrast to these widely expressed genes, a small subset, notably *MlWHy2* and *MIDehydrin5*, exhibited highly restricted expression patterns. This indicates that these genes may perform more specialized functions.

## 4. Discussion

*M. laxiflora*, a pioneer species in the hydrologically extreme water-level fluctuation zone of the Yangtze River, represents a critical model for studying flooding tolerance mechanisms. As a key scientific challenge in ecological restoration, we present the first systematic genomic analysis of the *LEA* gene family in this species. Through integrated bioinformatics approaches, we characterized the evolutionary dynamics and functional divergence of LEA proteins, revealing their putative roles in flooding adaptation strategies.

### 4.1. The Significant Expansion and Adaptive Evolution of LEA Gene Family

The LEA gene family is ubiquitous in plants and plays crucial roles in regulating growth and development under abiotic stress [16,17,18]. In *M. laxiflora*, we identified 31 *MlLEA* genes. Chromosomal localization revealed their distribution across all 12 chromosomes (Figure 1), with phylogenetic analysis classifying them primarily into the LEA_2, dehydrin, and WHy subfamilies (Table 1; Figure S1). These subfamilies exhibit evolutionarily conserved functions in osmotic stress responses [11,19,20,21]. Recent comparative genomic analyses have elucidated the evolutionary trajectories of the *LEA* gene family. In terrestrial plants, this family has undergone significant expansion via whole-genome duplication (WGD) and tandem duplication events. These genomic innovations correlate strongly with plant adaptation to xeric environments following terrestrial colonization [22]. Notably, dehydrin genes form clustered tandem arrays (Figure 5A), with their promoter regions enriched in *cis*-elements responsive to methyl jasmonate (MeJA), abscisic acid (ABA), low temperature, and light (Figure 4). This suggests that functional diversification post-duplication may enhance adaptation to periodic abiotic stresses [23,24,25]. These genomic features indicate that *M. laxiflora*’s duplication events likely prioritize hypoxia adaptation, reflecting a species-specific evolutionary strategy.

### 4.2. Diversified Regulation of Promoter Cis-Acting Elements

A promoter analysis revealed that *MlLEA_2* genes harbor anaerobic-inducible elements (e.g., ARE motifs), with significant enrichment observed in *MlLEA_2-1* and *MlDehydrin4* (Figure 4). These *cis*-elements may be activated through plant hypoxia signaling pathways (e.g., HIF-1) to mitigate root hypoxia damage during flooding [26]. LEA gene expression is primarily regulated by the ABA signaling cascade, with core transcription factors (e.g., ABI3/ABI5) directly binding the ABRE motifs in promoter regions [27]. This regulatory mechanism is evolutionarily conserved, as evidenced by exogenous ABA-inducing *ZmLEA_4* expression to enhance abiotic stress tolerance in maize [28]. In *M. laxiflora*, ABRE enrichment in *MlLEA_2-10* and *MlLEA_5-1* promoters confirms the central role of ABA signaling in stress perception and LEA transcriptional control [28,29]. Hormone crosstalk elements (e.g., MeJA- and GA-responsive motifs) may integrate multiple environmental signals through transcriptional regulatory networks, enabling spatiotemporal precision in stress responses [30]. Notably, *MlLEA_2-3* and *MlLEA_2-6* promoters co-localize low-temperature (LTR) and drought-responsive (MBS) *cis*-elements, suggesting potential dual stress-protection functions during compound stressors in riparian zones (e.g., winter flooding with concomitant hypothermia).

### 4.3. Relationship Between Expression Pattern and Flooding Stress Response

Studies have shown that LEA proteins enhance plant stress resistance through multiple molecular mechanisms by (1) functioning as molecular chaperones to prevent stress-induced protein misfolding and aggregation (e.g., *Arabidopsis AtLEA4-5* stabilizes client proteins via hydrophobic domain interactions) [31]; (2) maintaining membrane integrity through phospholipid bilayer binding (e.g., rice *OsLEA3-1* anchors to membranes, reducing drought-induced lipid peroxidation) [32]; and (3) Synergizing with antioxidant systems to scavenge reactive oxygen species (ROS) and mitigate oxidative damage [33].

Under continuous flooding stress, multiple *MlLEA* genes exhibited distinct temporal expression dynamics. For instance, *MlLEA_2-1* and *MlDehydrin4* showed rapid up-regulation (>5-fold) within 6–12 h (Figure 6A). As established molecular chaperones, these proteins likely mitigate hypoxia-driven protein misfolding by stabilizing native conformations during early stress phases [34,35]. This rapid response resembled the early dehydration-induced expression pattern of *A. thaliana AtLEA4-5* [36]. However, the activation threshold of *M. laxiflora* LEA genes was lower, potentially reflecting pre-adaptation to periodic flooding. Notably, *MILEA_3-1* and *MILEA_5-1* maintained high expression levels at 48 h but rapidly downregulated during the recovery phase (RR12) (Figure 6A). This suggests that prolonged flooding induces sustained cellular protection needs, while rapid gene expression resetting post-stress avoids energy waste. This dynamic regulation aligns with the expression attenuation observed in sweet potato [10,37,38], yet *M. laxiflora* exhibits a more rapid recovery, which may correlate with frequent water-level fluctuations in its habitat. *MlLEA* genes exhibited significant tissue-specific expression divergence (Figure 6B). For instance, *MlLEA_2-1* showed root-specific high expression, whereas *MlLEA_5-1* was predominantly expressed in roots. Functional predictions suggest that root-localized LEA proteins (e.g., *MlDehydrin*) [39,40] may maintain plasma membrane integrity via phospholipid bilayer anchoring, while leaf-enriched LEA proteins (e.g., *MlLEA_5-1*) potentially cooperate with antioxidant enzymes (e.g., SOD/POD) to mitigate photoinhibition damage [12,41]. This organ-specific functional partitioning underscores *M. laxiflora’s* adaptation to riparian ecotones through compartmentalized stress-response strategies.

## 5. Conclusions

This study presents a systematic genomic analysis of the *M. laxiflora LEA* gene family. We identified 31 *MlLEA* genes classified into 9 subfamilies based on protein sequence phylogeny, distributed across all 12 chromosomes. Our findings reveal adaptive evolutionary mechanisms underlying *LEA* family diversification in the Yangtze River’s water-level fluctuation zone, characterized by (1) significant expansion of the *MlLEA_2* subfamily with retention of conserved hydrophilic domains, enabling periodic flooding-drought adaptation through neofunctionalization; (2) promoter architecture enriched with anaerobic-inducible, ABRE, and hormone-crosstalk cis-elements, forming integrated environmental sensing networks; and (3) organ-specific functional divergence: root-localized genes (e.g., *MlDehydrin*) maintain membrane integrity via phospholipid anchoring, while leaf-predominant genes (e.g., *MlLEA_5-1*) mitigate photoinhibition through antioxidant coordination. These results provide molecular targets for ecological restoration in the Three Gorges Reservoir riparian zone and establish a framework for deciphering plant stress resilience mechanisms.

## Figures and Tables

**Table 1:** Structural domain identification and family classification of *LEA* gene family in *M. laxiflora*.

Gene Name	Domain Name	Domain ID	Start	End	Length	E-Value
*MlDehydrin1*	Dehydrin	PF00257	64	123	60	1.56 × 10^−7^
	Dehydrin	PF00257	152	226	75	1.26 × 10^−6^
*MlDehydrin2*	Dehydrin	PF00257	102	161	60	1.91 × 10^−9^
*MlDehydrin3*	Dehydrin	PF00257	93	153	61	2.78 × 10^−7^
	Dehydrin	PF00257	182	255	74	1.92 × 10^−3^
*MlDehydrin4*	Dehydrin	PF00257	167	231	65	3.85 × 10^−7^
*MlDehydrin5*	Dehydrin	PF00257	15	144	130	1.39 × 10^−12^
*MlDehydrin6*	Dehydrin	PF00257	24	247	224	1.42 × 10^−9^
*MlLEA_1-1*	lLEA_1	PF03760	156	222	67	5.65 × 10^−21^
*MlLEA_1-2*	lLEA_1	PF03760	9	72	64	2.67 × 10^−12^
*MlWHy1*	WHy	PF08267	25	124	100	3.67 × 10^−22^
*MlWHy2*	WHy	PF08267	267	366	100	5.79 × 10^−24^
*MlWHy3*	WHy	PF08267	91	190	100	7.51 × 10^−28^
	WHy	PF08267	209	331	123	5.98 × 10^−21^
*MlLEA_3-1*	LEA_3	PF03242	1	89	89	1.16 × 10^−30^
*MlLEA_5-1*	LEA_5	PF00477	2	87	86	2.61 × 10^−26^
*MlLEA_6-1*	LEA_6	PF10714	15	74	60	3.53 × 10^−21^
*MlSMP1*	SMP	PF04927	21	53	33	7.80 × 10^−4^
*MlLEA_1-3*	lLEA_1	PF03760	1	66	66	3.60 × 10^−5^
*MlLEA_2-10*	LEA_2 Superfamily	PF03168	94	142	49	9.28 × 10^−3^
*MlLEA_2-11*	LEA_2 Superfamily	PF03168	7	192	186	2.18 × 10^−39^
	LEA_2 Superfamily	PF03168	223	414	192	2.77 × 10^−24^
*MlLEA_2-1*	LEA_2	PF03168	97	202	106	5.73 × 10^−5^
*MlWHy4*	WHy	PF08267	240	323	84	6.90 × 10^−16^
	WHy	PF08267	41	139	99	1.55 × 10^−15^
*MlLEA_2-2*	LEA_2	PF03168	93	197	105	1.27 × 10^−3^
*MlLEA_2-3*	LEA_2	PF03168	180	283	104	4.04 × 10^−7^
*MlLEA_2-4*	LEA_2	PF03168	146	250	105	2.45 × 10^−8^
*MlLEA_2-5*	LEA_2	PF03168	78	182	105	2.22 × 10^−8^
*MlLEA_2-6*	LEA_2	PF03168	91	186	96	2.71 × 10^−8^
*MlLEA_2-12*	LEA_2 super family	PF03168	46	224	179	8.20 × 10^−12^
*MlLEA_2-13*	LEA_2 super family	PF03168	66	236	171	1.09 × 10^−7^
*MlLEA_2-7*	LEA_2	PF03168	159	263	105	2.09 × 10^−3^
*MlLEA_2-14*	LEA_2 super family	PF03168	20	211	192	2.75 × 10^−22^
*MlLEA_2-8*	LEA_2	PF03168	138	241	104	5.22 × 10^−7^
*MlLEA_2-9*	LEA_2	PF03168	193	292	100	6.14 × 10^−3^

**Table 2:** 31 LEA family genes and related information of *M. laxiflora*.

Gene Name	Gene ID	Subfamily	Subcellular Location	CDS Size	Protein Size	MW/KDa	pI	Instability Index	Aliphatic Index	GRAVY
*MlDehydrin1*	Myl03g00992.1	Dehydrin	Chloroplast	696	231	24.87	9.54	25.44	37.6	−1.392
*MlDehydrin2*	Myl08g00301.1	Dehydrin	Chloroplast	519	172	19.1	6.46	55.21	42.6	−1.369
*MlDehydrin3*	Myl03g00991.1	Dehydrin	Chloroplast	783	260	27.71	9.43	25.9	36.7	−1.326
*MlDehydrin4*	Myl03g00338.1	Dehydrin	Chloroplast	750	249	27.82	5.47	55.85	49.9	−1.378
*MlDehydrin5*	Myl03g00989.1	Dehydrin	Chloroplast	627	208	22.14	6.71	38.97	30.4	−1.378
*MlDehydrin6*	Myl08g00106.1	Dehydrin	Chloroplast	744	247	26.53	6.68	29.65	51.1	−1.002
*MlLEA_1-1*	Myl09g00459.1	LEA_1	Chloroplast	798	265	29.02	9.52	46.03	70	−0.579
*MlLEA_1-2*	Myl06g01621.1	LEA_1	Chloroplast	459	152	15.76	9.13	19.26	44.5	−0.774
*MlLEA_1-3*	Myl12g00406.1	LEA_1	Chloroplast	231	76	8.694	6.84	35.63	63.3	−1.064
*MlLEA_2-1*	Myl02g01162.1	LEA_2	Chloroplast	693	230	26.31	9.56	57.55	84.8	−0.27
*MlLEA_2-2*	Myl04g01868.1	LEA_2	Chloroplast	666	221	25.31	9.35	36.44	91.7	−0.196
*MlLEA_2-3*	Myl04g02032.1	LEA_2	Chloroplast	927	308	34	9.18	42.85	85	−0.207
*MlLEA_2-4*	Myl05g00215.1	LEA_2	Chloroplast	1110	369	40.42	10.25	48.67	87.7	−0.146
*MlLEA_2-5*	Myl06g01866.1	LEA_2	Chloroplast	657	218	24	9.41	32.09	95.2	−0.008
*MlLEA_2-6*	Myl08g01177.1	LEA_2	Chloroplast	570	189	21.01	9.55	50.03	114	0.138
*MlLEA_2-7*	Myl09g01907.1	LEA_2	Chloroplast	864	287	31.94	9.32	44.02	83.8	−0.249
*MlLEA_2-8*	Myl10g02084.1	LEA_2	Chloroplast	843	280	30.56	10.09	41.04	80.1	−0.147
*MlLEA_2-9*	Myl12g00097.1	LEA_2	Chloroplast	957	318	35.7	8.5	61.28	81.5	−0.297
*MlLEA_3-1*	Myl09g02902.1	LEA_3	Chloroplast	309	102	11.19	7.93	43.05	69.8	−0.46
*MlLEA_5-1*	Myl07g01997.1	LEA_5	Chloroplast	294	97	10.46	8.12	63.95	45.3	−1.31
*MlLEA_6-1*	Myl03g00855.1	LEA_6	Chloroplast	231	76	8.08	5.58	56.29	28.3	−378
*MlWHy1*	Myl12g00170.1	WHy	Chloroplast	456	151	16.37	4.68	16.03	108	0.148
*MlWHy2*	Myl04g00578.1	WHy	Chloroplast	1182	393	43.57	5.84	38.55	76.3	−0.475
*MlWHy3*	Myl11g00251.1	WHy	Chloroplast	1107	368	40.96	5.13	20.59	99.3	−0.22
*MlWHy4*	Myl04g00969.1	WHy	Chloroplast	1116	371	40.79	9.67	34.73	103	0.095
*MlSMP1*	Myl09g00630.1	SMP	Chloroplast	792	263	28.72	9.81	42.05	81.6	−0.327
*MlLEA_2-10*	Myl01g00162.1	LEA_2 superfamily	Chloroplast	654	217	25.01	9.9	47.78	99.2	−0.236
*MlLEA_2-11*	Myl02g00806.1	LEA_2 superfamily	Chloroplast	1263	420	45.91	9.09	22.82	115	0.248
*MlLEA_2-12*	Myl08g01489.1	LEA_2 superfamily	Chloroplast	681	226	25.37	8.35	47.41	94	−0.078
*MlLEA_2-13*	Myl09g00692.1	LEA_2 superfamily	Chloroplast	711	236	26.13	9.36	43.07	75.9	−0.168
*MlLEA_2-14*	Myl10g01528.1	LEA_2 superfamily	Chloroplast	648	215	23.45	9.51	27.41	116	0.237

## Data Availability

The whole genome sequencing data of M. laxiflora Download (https://figshare.com/articles/dataset/Myricaria_laxiflora_genome_and_annotation_files/25375366); Transcriptome data download (PRJNA1164124 and PRJNA840865). All data in this manuscript are based on genome and transcriptome analysis, and no new data are generated.

## Supplementary Materials

The following supporting information can be downloaded at https://www.mdpi.com/article/10.3390/genes16070763/s1; Figure S1: Sequence alignment of LEA gene family subfamily in Myricaria laxiflora. (A) Sequence alignment of Dehydrin subfamily. (B) Sequence alignment of LEA_1 subfamily. (C) Sequence alignment of LEA_2 subfamily. (D) Sequence alignment of LEA_2 superfamily subfamily. (E) Sequence alignment of WHy subfamily. (F) Sequence alignment of LEA_3, LEA_5, LEA_6 and SMP subfamily, the yellow marker is the domain sequence.
