# Supplementary material for: Genome-Wide Identification of the LEA Gene Family in Myricaria laxiflora and Its Responses to Abiotic Stress
Source: Genes (Basel). 2025 Jun 29;16(7):763. doi: 10.3390/genes16070763 (PMC12294544; doi:10.3390/genes16070763)
Supplement: Supplementary file 1 [file genes-16-00763-s001.zip › genes-3716859-supplementary.pdf]

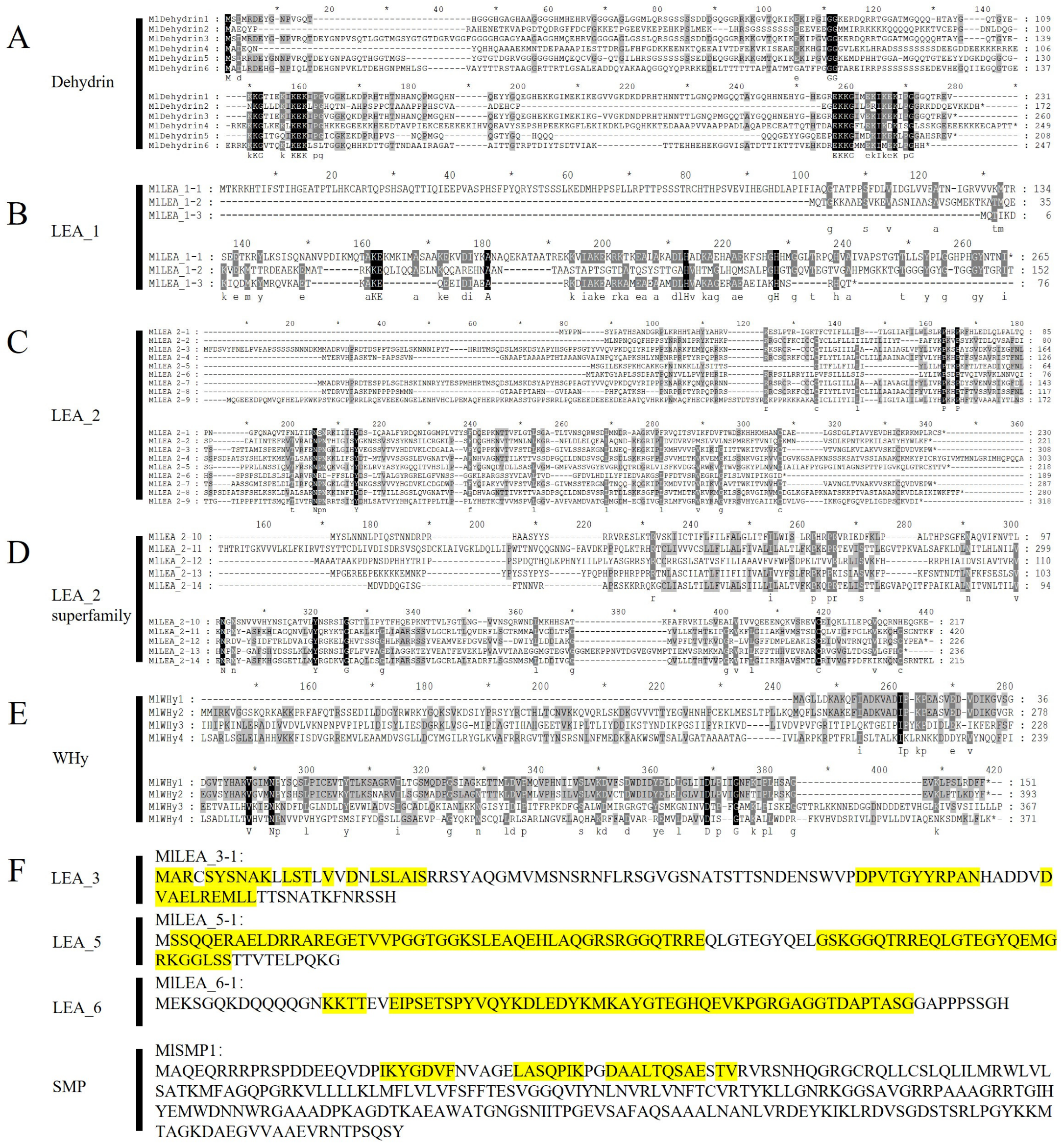

Figure S1. Sequence alignment of LEA gene family subfamily in *Myricaria laxiflora.* (A) Sequence alignment of Dehydrin subfamily. (B) Sequence alignment of LEA_1 subfamily. (C) Sequence alignment of LEA_2 subfamily. (D) Sequence alignment of LEA_2 superfamily subfamily. (E) Sequence alignment of WHy subfamily. (F) Sequence alignment of LEA_3, LEA_5, LEA_6 and SMP subfamily, the yellow marker is the domain sequence.
